# A rare pediatric case of thoracic spine giant cell tumor: Clinical implications and surgical strategies

**DOI:** 10.1016/j.ijscr.2025.110890

**Published:** 2025-01-13

**Authors:** Ibrahim Fathallah, Ayham Qatza, Ahmed Al-Talep, Abd Alrhman Alajrd, Mohammad Alfattal, Ahmad Alhamoud

**Affiliations:** a Faculty of Medicine, Homs University, Homs, Syria; b Faculty of Medicine, Damascus University, Damascus, Syria; c Department of Neurosurgery, Damascus Hospital, Damascus, Syria; d Faculty Of Medicine, Al-fourat university, Deir ez-Zor, Syria; e Faculty of Medicine, Hama University, Hama, Syria

**Keywords:** Giant cell tumor, Benign tumor, Thoracic spine, Case report

## Abstract

**Introduction and clinical importance:**

Giant cell tumor (GCT) is a rare benign bone tumor that usually affects skeletally adult people. While it usually appears in the epiphyseal parts of long bones, it is very rare in the spine, particularly the thoracic spine, especially in pediatric patients.

**Case presentation:**

An 11-year-old female presented with progressive lower extremity weakness, accompanied by localized back pain and urinary urgency, diagnosed with GCT in the thoracic spine. Magnetic resonance imaging (MRI) revealed a large, highly vascularized tumor involving the D6 and D7 vertebrae, with significant spinal cord compression. The patient underwent an urgent surgical resection of the tumor. Spinal stabilization was performed using a pedicle screw and rod construct. Postoperatively, the patient demonstrated significant improvement in muscle strength and functional recovery.

**Clinical discussion:**

This case highlights the rarity of thoracic spine GCTs and emphasizes the critical role of MRI in diagnosis. It underscores the necessity of prompt surgical resection and spinal stabilization for patients with spinal GCT to ensure effective management and prevent neurological complications.

**Conclusion:**

This report emphasizes the rarity of thoracic spine GCTs in pediatric patients, advocating for heightened clinical awareness, MRI use for early detection, and standardized management protocols to improve outcomes. Further research and education among clinicians are essential.

## Highlights

• A rare case of giant cell tumor (GCT) in the thoracic spine of a pediatric patient.
• MRI imaging was essential and crucial in the diagnostic process.
• Urgent surgery is the gold standard for this type of tumor.
• The importance of early diagnosis and standardized treatment protocols.

## 1 Introduction

Giant cell tumor (GCT) of the bone is a benign and rare tumor that only represents 4 % of all primary bone tumors [1]. Despite being considered benign, it can present as a locally invasive lesion, resulting in extensive bone and soft tissue destruction. GCTs commonly affect skeletally mature patients aged 20 to 40 years, making their occurrence in the immature skeleton exceptionally rare and poorly documented in the literature [1,2]. Although GCT can affect people of any ethnic origin, it is more common in Chinese and South Indian populations where it amounts to 20–30 %]1[. GCTs typically affect the epiphyseal regions of the long bones, especially at the distal femur and proximal tibia, with over 50 % located near the knee joint [1,3]. GCTs represent less than 10 % of spinal tumors, primarily located in the sacrum, while instances in the thoracic spine are rarely documented in literature [2,4]. About 2 % to 5 % of the GCT cases have been discovered in the vertebra above the sacrum [4] . Acute onset of pain may be linked with a pathologic fracture, which can occur at diagnosis in approximately 10–12 % of cases. Neurological complications may be associated with spinal GCT [1]. This paper describes a rare case of GCT, highlighting the tumor's rare location and the patient's age at injury, and provides a comprehensive overview of the diagnostic strategies, surgical approaches, and outcomes to raise awareness of this rare disorder.

This work is also reported in line with SCARE criteria, thereby increasing the report's transparency and quality [5].

Case Presentation.

**Fig. 1:**
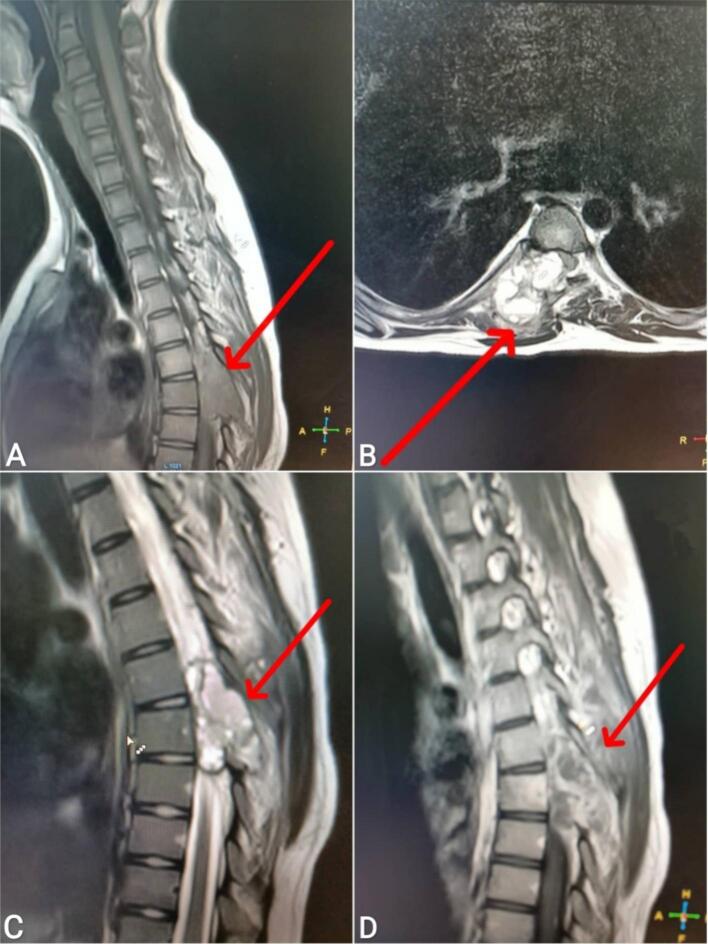
Non-contrast (images A, B, and C) and contrast (image D) magnetic resonance imaging revealed a tumorous lesion at the level of the D6 and D7 vertebrae, measuring about 4 cm × 4 cm × 5 cm. The lesion showed high signal intensity on T2-weighted images, low signal intensity on T1-weighted images, and heterogeneous enhancement post-contrast image, with erotion of the D6 posterior arch and the right pedicle, causing spinal cord compression. (A: Sagittal section of T1-weighted sequence, B: Axial section of T2-weighted sequence, C: Sagittal section of T2-weighted sequence, D: Sagittal section of T1-weighted sequence).

**Fig. 2:**
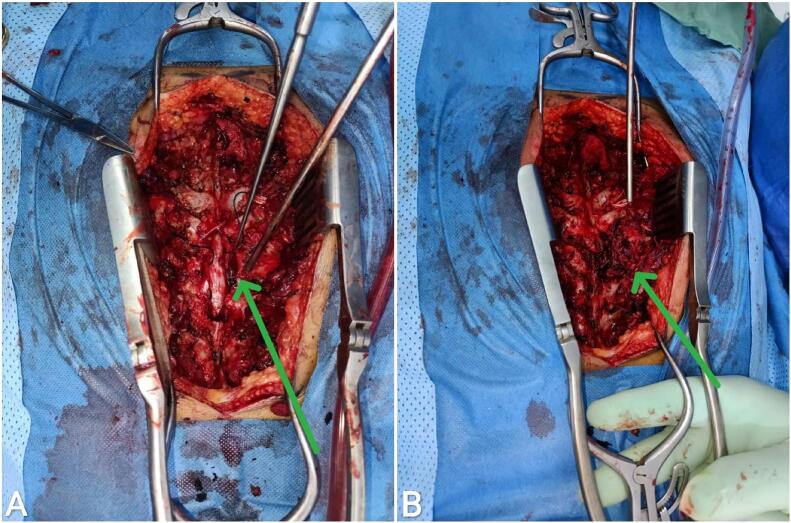
Intraoperative images demonstrate that the lesion eroded the right lamina, facet joint, and rib junction (image B). Following complete tumor resection, a defect in the vertebral body was visibled, with the spinal cord having been relieved from compression (image A).

**Fig. 3:**
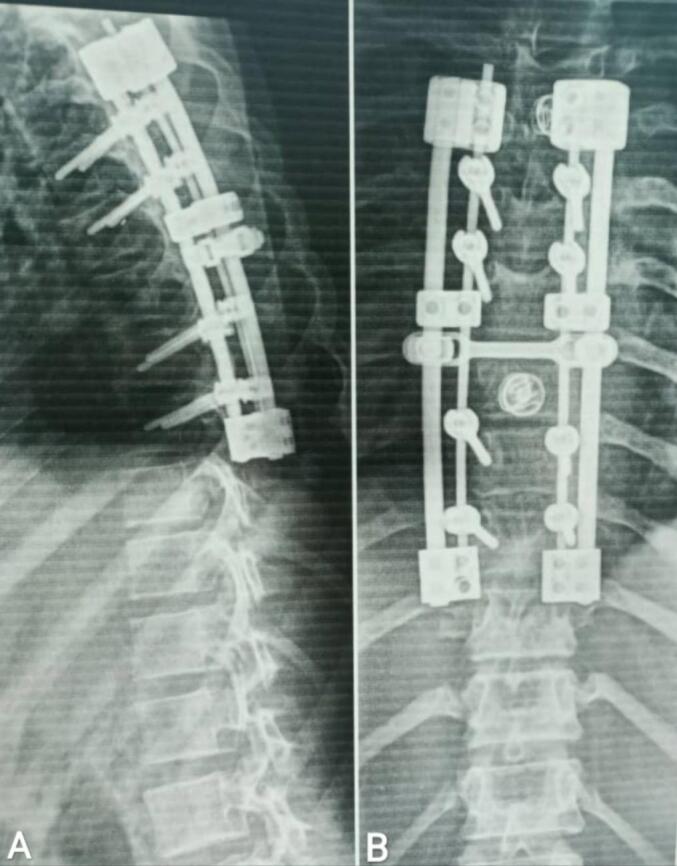
Anteroposterior (image B) and lateral (image A) X-rays of the thoracic spine reveal a transpedicular fixation device comprising eight pedicle screws at T5, T6, T8, and T9 levels, along with connectors and four rods. Lateral mass screws were utilized due to the unavailability of pediatric devices.

**Fig. 4:**
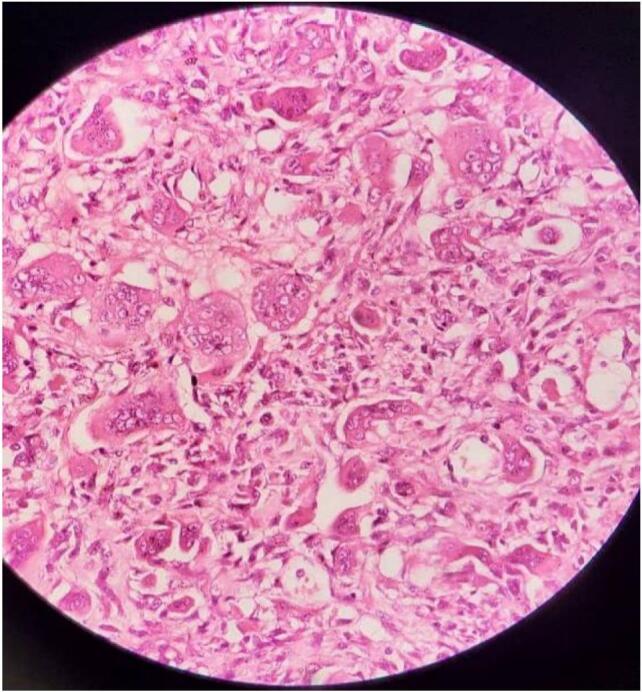
Histological examination showed bone trabecula and a tumor comprised mainly of multinucleated giant osteoclasts and spindle-shaped mononuclear macrophage-like cells. The stroma contains blood vessels and diffuse hemorrhagic regions, with no signs of sarcomatous change.

An 11-year-old female patient presented to the emergency department with progressive bilateral lower limb weakness, more severe on the right side, localized pain, and segmental distribution over one week. She was unable to stand for approximately three days and reported urinary urgency and straining. The patient had no history of trauma, congenital disorders, or familial illnesses, and denied previous surgeries or medication use. Vital signs and blood tests were within normal limits. Physical examination revealed proximal muscle weakness in both lower limbs (2/5 right thigh and 3/5 left thigh), along with hyperactive patellar and achilles reflexes, and decreased abdominal reflexes. Following the physical examination, non-contrast and contrast magnetic resonance imaging (MRI) revealed a tumorous lesion at the level of the D6 and D7 vertebrae, measuring about 4 cm × 4 cm × 5 cm. The lesion showed high signal intensity on T2-weighted images, low signal intensity on T1-weighted images, and heterogeneous enhancement post-contrast image, with erotion of the D6 posterior arch and the right pedicle, causing spinal cord compression. [Fig. 1]. Based upon the neurologic symptomatology and radiological findings, surgical excision was recommended, with the patient's consent obtained and medical consultations prior to the procedure revealing no objections. The surgery was performed two days after imaging. A neurosurgery specialist performed tumor resection, revealing the mass to be fragile, bleeding, and highly vascularized from a subcostal artery, eroding the bone [Fig. 2]. Spinal stabilization was achieved using eight pedicle screws, rods, and a bridge, with cervical screws employed due to the unavailability of pediatric screws [Fig. 3]. Regarding the difficulties in using screws in the child, the decision was challenging due to the unavailability of pediatric-specific screws. Cervical screws were used and reinforced with adult-sized fixation rods using a connecting device. In addition, there were numerous anatomical challenges during the child's surgery, including the need to minimize bleeding, the mismatch between the size of surgical instruments and the child's size, the small size of muscle mass and bony elements, and the proximity of anatomical structures, which posed challenges during fixation. A biopsy of the tumor was taken after excision and sent for pathological examination, which showed bone trabeculae and a tumor comprised mainly of multinucleated giant osteoclasts and spindle-shaped mononuclear macrophage-like cells. The stroma contains blood vessels and diffuse hemorrhagic regions, with no signs of sarcomatous change [Fig. 4]. Differential diagnosis initially raised suspicion of spinal tuberculosis, which was excluded by histopathological examination. Based on the aforementioned histological findings, the diagnosis of GCT in the seventh thoracic vertebra was made. Postoperatively, the patient received dexamethasone at a dosage of 2.5 mg twice daily (0.15 mg/kg based on a weight of 35 kg). Within 48 h, a significant muscle strength improvement was observed, enabling ambulation with assistance and the commencement of early physical therapy. One week post-discharge, the patient ambulated independently with full bladder control. Sutures were removed after two weeks, with continued physical therapy, and The patient was followed up after one and three months, respectively, with a normal neurological examination and no complications, recurrence of the tumor, or need for further intervention. An MRI was performed after six months as a follow-up, which was normal.

## 2 Discussion

GCT of bone is a rare condition with an annual incidence of approximately 1.3 per million, considered a primary benign but locally aggressive tumor [6,7]. Females are slightly more likely than males, and the incidence is highest within the aged 30 to 40 [7,8]. The biologic range of GCT is broad, encompassing hidden benign, highly frequent, and even metastatic malignant propensity [9]. Between 2.7 % and 6.5 % of all GCTs in bone are located in the spine [10]. The etiology of GCT is not fully understood, but a 20q11 amplification and over-expression of p53 may increase the likelihood of a GCT occurring [[11], [12], [13]]. Patients with spinal GCT commonly complain of pain, with up to 72 % of them having neurological disorders as a consequence of compression of the spinal cord and/or nerve roots [14]. The patient in the present case suffered from widespread, segmental pain and urinary retention due to a neurological disorder. Radiographs and MRIs are the most common imaging modalities for diagnosing GCT. Spinal GCT appears on radiographs as an expansile osteolytic lesion with multiple degrees of vertebral body destruction [15]. While MRI distinguishes these lesions from surgical complications, including infections or infectious spondylitis [16]. In the present case, an MRI showed a tumorous lesion compressing the spinal cord between the sixth and seventh thoracic vertebrae. Surgery is the preferred treatment for removing GCT, decompressing neural structures, and spinal stabilization. The procedure carries a significant risk of surgical morbidity, but if the tumor is resectable, complete surgical removal of the tumor, including a margin of healthy tissue, is associated with reduced recurrence and prolonged disease-free survival [7,14,17]. Pediatric patients showed lower overall and progression-free survival rates than adults [18]. Given the challenges in achieving complete excision of spinal GCTs and the high risk of local recurrence, various adjuvant therapies, including radiation, bisphosphonates, interferon-alpha, embolization, and denosumab, have been employed to reduce recurrence rates, survival rate following local recurrence with/without radiation was 91 % and 89 %, with/without embolization was 91 % and 86 %, and with/without local adjuvants was 88 % and 92 %. In regard to surgical treatment, the tumor recurred in 71 % of patients who had incomplete resections, but in 8 % of patients who had complete resections [14]. The Key items that need to be considered in surgical hardware selection are hardware placement, local infection state, duration of hardware exposure, and evidence of hardware loosening. However, the final outcome of treatment is mostly based on the surgeon's clinical observations and personal expertise [19]. Acute management of children spinal cord tumors typically includes surgical excision, chemotherapy, and/or radiation therapy. When a complete resection is not possible, radiation and chemotherapy are usually added. However, the treatment is not consistent [20] . In general, GCTs should be handled on an individual basis. Cooperation between spine surgeons, medical oncologists, and radiation oncologists is essential to choosing the proper treatment strategy for each patient [14].

## 3 Conclusion

This report underscores the rarity of GCTs in the thoracic spine of pediatric patients, emphasizing the need for heightened clinical awareness due to the risk of neurological compromise. Clinicians must maintain a high suspicion for GCTs in children with unexplained back pain and neurological deficits. In addition, the importance of MRI for early detection and intervention is highlighted. The paper advocates for standardized management protocols, including urgent surgical strategies. Future research should refine diagnostic criteria and treatment guidelines to enhance outcomes.

## List of abreviations

GCT: Giant cell tumor
MRI: Magnetic resonance imaging

## CRediT authorship contribution statement

Ibrahim Fathallah: Writing – review & editing, Writing – original draft, Data curation.

Ayham Qatza: Writing – review & editing, Writing – original draft.

Ahmed Al-Taleb: Writing – review & editing, Writing – original draft.

Abd Alrhman Alajrd: Writing – review & editing, Writing – original draft.

Mohammad Alfattal: Writing – review & editing, Writing – original draft.

Ahmad Alhamoud: Writing – review & editing, Supervisor

Ibrahim Fathallah: submitted the final manuscript.

All authors read and approved the final manuscript.

## Ethics approval and consent to participate

Ethics clearance was not necessary since the University waives ethics approval for publication of case reports involving no patients' images, and the case report is not containing any personal information. The ethical approval is obligatory for research that involve human or animal experiments.

## Consent for publication

Written informed consent was obtained from the patient's parents/legal guardian for publication and any accompanying images. A copy of the written consent is available for review by the Editor-in-Chief of this journal on request.

## Funding

The author(s) received no financial support for the research, authorship, and/or publication of this article.

## Declaration of competing interest

The author(s) declared no potential conflicts of interest with respect to the research, authorship, and/or publication of this article.

## Data Availability

Data sharing not applicable to this article as no datasets were generated or analyzed during the current study.
